# The remineralizing ability of self-assembling peptide P11-4, 2% arginine enriched sodium fluoride and functionalized tri calcium phosphate fluoride varnishes in treatment of white spot lesions – a randomized controlled trial

**DOI:** 10.1038/s41405-025-00353-5

**Published:** 2025-07-27

**Authors:** Bardis Salah Abd Elaziz, Dina Hamdy, Mona Galal, Nagwa Mohammed Ali Khattab

**Affiliations:** 1 Pediatric Dentistry & Dental Public Health, Faculty of Dentistry, Modern University for Technology and Information (MTI), Cairo, Egypt; 2 Ain Shams University Faculty of Dentistry, Cairo, Egypt; 3 Pediatric Dentistry and Dental Public Health Faculty of Dentistry- Ain Shams University Cairo, Cairo, Egypt; 4 Operative Dentistry Faculty of Dentistry- Ain Shams University Cairo, Cairo, Egypt

**Keywords:** Minimal intervention dentistry, Paediatric dentistry

## Abstract

**Background:**

White spot lesions (WSLs) is a caries lesion distinguished by white opacity due to subsurface enamel demineralization. These lesions resulting from a disparity between detrimental and protective factors and can continue to demineralize if untreated. Early diagnosis and remineralization can reverse WSLs, supporting minimally invasive dental care.

**Aim:**

To compare and assess the color of WSLs through spectrophotometer after application of Self-assembling peptide P11-4, 2% Arginine Enriched Sodium Fluoride varnish and Functionalized Tri Calcium Phosphate Fluoride varnish.

**Methods:**

This randomized controlled triple-blind clinical trial enrolled pediatric participants (ages 8–12 years) presenting with moderate caries risk as defined by CAMBRA (Caries Management By Risk Assessment) criteria, with visible white spot lesions (WSLs) on the labial surfaces of maxillary permanent anterior teeth, classified as ICDAS scores 1 or 2. A total of 39 WSLs were incorporated into the study and randomly allocated into one of the three groups 13/each. Group I: Self-assembling peptide P11-4 varnish (study group), Group II: Arginine-enriched Sodium Fluoride varnish (study group) and Group III: Tri Calcium Phosphate Fluoride varnish (positive control group). The color change ΔE of each WSL was quantified using a spectrophotometer at the baseline. Dimensions of WSLs were assessed by digital photography, remineralizing agents were applied then patients were recalled for further applications after 3 and 6 months and for WSLs assessment after 3, 6 and 9 months.

**Results:**

All groups demonstrated progressive color improvement over time. At 3 months, Group I showed the least improvement (ΔE = 16.39 ± 3.04), followed by Group III (14.80 ± 3.11) and Group II (14.06 ± 4.46). By 9 months, Group II achieved (ΔE = 9.37 ± 3.79), and Group III (9.15 ± 2.74) surpassing Group I (12.21 ± 3.03). so both Group II and III ultimately outperformed Group I in color correction by the study’s end. Group II achieved significantly greater WSL reduction (14.98 ± 7.55%) compared to both Group I (27.93 ± 8.98%) and Group III (22.32 ± 8.61%), with no significant difference observed between Groups I and III.

**Conclusions:**

Although all the three tested materials demonstrated an enhancement in the color and dimension of WSLs, 2% of Arginine Enriched Sodium Fluoride showed the best results followed by Functionalized Tri Calcium Phosphate Fluoride varnish, while self-assembling peptide P11-4 showed the lowest results.

## Background

White spot lesion is characterized as a white opacity resulting from subsurface enamel demineralization on smooth surfaces. The white look is attributed to alterations in the optical light scattering characteristics of the decalcified enamel. Consequently, early treatment including remineralization instead of restoring such WSLs is considered a crucial element of minimally invasive dentistry [1].

White spot lesions are triggered by pH changes resulting from the metabolic activities of bacteria present in the biofilm. These variations induce demineralization and remineralization. Consequently, the demineralization of tooth hard tissues occurs, resulting in the initiation of dental caries [2, 3].

In recent decades, various treatments, in addition to the necessity for superior oral hygiene, have been implemented to prevent the formation of white spot lesions (WSL), particularly the topical use of fluoride, which serves as the benchmark for other remineralization systems [4, 5]. The optimal remineralizing substance must facilitate the transfer of calcium and phosphate into the lesion. Fluoride agents can remediate initial lesions, although their impact is merely superficial [6]. This may lead to the remineralization of the porous surface layer, resulting in the obstruction of enamel pores, hindering ionic exchange at the surface enamel, and obstructing the remineralization of the lesion’s body, so challenging the achievement of complete remineralization [6].

One of the recent advances in remineralization is self-assembling peptides, produced by Credentis AG, Windisch, Switzerland, and devoid of human or animal-derived components. Biocompatibility testing performed according to ISO 10993 standards has confirmed that peptide P11-4 demonstrates no evidence of cytotoxicity in biological systems [7]. In a randomized controlled trial by Bröseler et al. [8], researchers evaluated the effectiveness of combining P11-4 peptide with fluoride varnish for white spot lesion treatment. Their findings revealed significantly greater reduction in early caries lesion size with P11-4 compared to fluoride varnish monotherapy.

L-arginine, an endogenous component of human saliva, is present in the oral cavity at physiological concentrations averaging 50 μM. Oral microbiota including Streptococcus, Lactobacillus, and Treponema species catabolize this amino acid through the arginine deiminase pathway, producing ornithine, ammonia, and CO₂. The resulting ammonia release elevates local pH, potentially contributing to oral ecological homeostasis [9]. Prior studies indicated that including arginine into dentifrices improved white spot lesions in comparison to non-arginine toothpastes. Furthermore, the integration of arginine with 5% sodium fluoride varnish produced a synergistic effect with increased F/Arg release [10, 11].

Modern fluoride varnishes incorporating bioactive tricalcium phosphate compounds (e.g., Clinpro™ 5% varnish) exhibit enhanced efficacy in managing incipient carious lesions. These formulations function through a unique mechanism where a protective calcium ion matrix forms during product formulation. Subsequent exposure to the oral environment triggers controlled release of fluoride, calcium, and phosphate ions from this matrix, enabling targeted remineralization of demineralized enamel [12]. Previous research documented that TCP fluoride varnish has greater protection against a cariogenic challenge and showed more reduction in lesion depth when compared with varnish containing 5% sodium fluoride alone [13, 14].

Demineralization modifies the physiological reflectivity of enamel, the disparity in refractive index between normal enamel and the demineralized region produces a milky white opaque look, distinctly identifiable from the adjacent intact enamel, facilitating the assessment of enamel color alterations caused by WSL treatment via spectrophotometric analysis [15–17].

The color of teeth can be assessed via a spectrophotometer on a CIELAB scale. The International Commission on Illumination, developed the CIELAB scale, which situates an object’s color inside a three-dimensional color space. The three axes are L*, a*, and b*. The coordinate L* quantifies an object’s lightness, ranging from 0 (absolute darkness) to 100 (pure white). Consequently, an elevated L* value indicates increased light reflectance of the object (CIE, 1978). The a* and b* coordinates measure color, representing an object’s location between red and green (a*) and yellow and blue (b*) [18].

Clinical dental photography is a helpful tool in assessment of percentage of WSLs. Recent computer programs are now used to analyze the digital images such as Adobe Photoshop and Auto CAD [19].

### Aim of the study

This randomized controlled clinical investigation evaluated chromatic changes and dimension of white spot lesions following treatment with three distinct modalities: self-assembling peptide therapy, 2% arginine-enriched sodium fluoride varnish, and tri-calcium phosphate fluoride varnish.

The study postulated the null hypothesis that no statistically significant differences would exist among the remineralization efficacies of: P11-4 self-assembling peptide, 2% arginine-enriched sodium fluoride varnish and functionalized tri-calcium phosphate fluoride varnish, when treating incipient carious lesions.

## Subjects, materials, and methods

### Ethical considerations

The study protocol was reviewed by the Research Ethics Committee and Institutional Review Board, Faculty of Dentistry, Ain Shams University, with reference number (FDASU-Rec ID 032107). Both verbal and written consent were obtained from the participant’s caregiver following an explanation of the study’s aims and a guarantee of complete data confidentiality. All caregivers and subjects were apprised of their ability to quit from the study at any time. Additionally, subjects were requested to fill out an assent form after receiving an appropriate age-specific description of the study methodology and objectives. All consent and assent documents were rendered into Arabic.

### Study design and settings

The investigation employed a randomized controlled trial design with three parallel treatment arms, utilizing equal allocation (1:1:1 ratio) across study groups. The trial protocol has been submitted at ClinicalTrials.gov PRS with ID NCT05127889. The study was conducted at the outpatient clinic of the Pediatric Dentistry and Dental Public Health department, Faculty of Dentistry, Ain Shams University.

### Sample size estimation

A power analysis was designed to have adequate power to apply a three-sided statistical test of the null hypothesis that there is no significant difference between tested groups. By adopting an alpha level of (0.05) a beta of (0.2) i.e., power = 80% and an effect size (d) of (1.30) calculated based on the results of previous studies [8, 20]; the predicted sample size (*n*) was a total of (33) WSLs. Sample size calculation was performed using G*Power version 3.1.9.7 [21].

The group’s sample was raised by 15% to consider attrition, resulting in a total inclusion of 39 WSLs / 13 per group.

### Eligibility criteria

Participants were screened using CAMBRA-based criteria: presence of active WSLs (disease indicator), adequate salivary function (protective factor), and no xerostomia (risk factor exclusion) [22].

#### Inclusion criteria

- Patients aged from 8 to 12 years old have WSLs on the labial surface of upper permanent anterior teeth with a score of 1 & 2 according to the ICDAS that don’t require operative intervention indicating moderate caries risk per CAMBRA criteria [22, 23].
- Patients who exhibited normal salivary flow (>0.7 mL/min stimulated) and no xerostomia, confirming adequate remineralization potential (protective factor).
- Patients free from any systemic, genetic or developmental abnormalities [23].

#### Exclusion criteria

- patients with high-risk CAMBRA indicators (e.g., xerostomia, frequent sugar exposure >3x/day), developmental enamel defects, or malocclusion impairing plaque control.
- Patients who received tetracyclines or any other medication known to stain teeth or causing xerostomia [1].
- Uncooperative patients [5].
- Patients with class II or III malocclusion [5].
- Mouth breathers [5].
- Participants having non- carious lesions (enamel hypoplasia, dental fluorosis, etc.) [23].

### Randomization and allocation

**Fig. 1:**
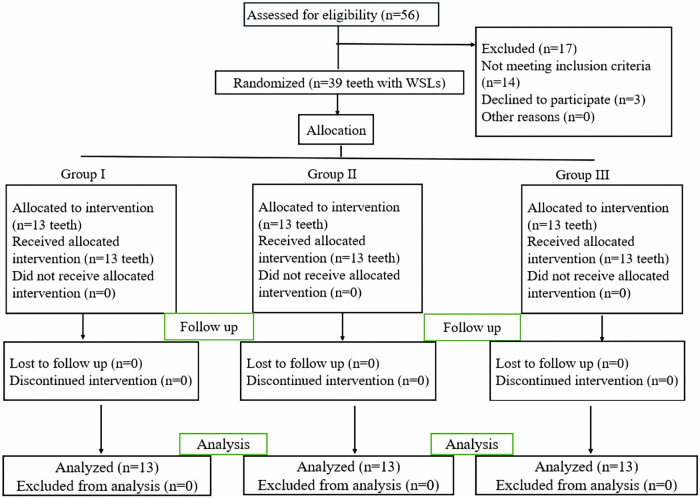
Flowchart depicting trial recruitment, randomization, allocation, and analysis during the follow-up period in accordance to CONSORT principles.

The research design adhered strictly to the Consolidated Standards of Reporting Trials (CONSORT) guidelines, with the study flowchart presented as Fig. 1. Each tooth exhibiting WSL was assigned a numerical designation from (1) to (39) and randomly distributed among the three experimental groups (13 teeth per group) by using IBM SPSS V26 (IBM, USA) statistical analysis software. Each tooth in each participant represented its own control. In the current study, randomization was dependent on two interrelated aspects, efficient sequence generation and unpredictable allocation concealment until the trial was completed. The allocation system was set up to enroll participants. All participant records were available only to the main investigator [24].

### Groupings

Group I: Self-assembling peptide P11-4 varnish (study group *n* = 13)

Group II: Arginine-enriched, Sodium Fluoride varnish (study group *n* = 13)

Group III: Tri Calcium Phosphate Fluoride varnish (positive control group *n* = 13).

#### Blinding

The investigation utilized a triple-blind design, maintaining blinding of participants, outcome assessors and statistician throughout all follow-up evaluations. Reliability assessments included calculation of Intraclass Correlation Coefficients (ICC) to determine both inter- and intra-examiner consistency. ICC for intra examiner reliability was 0.82 which indicated consistency of the examiner over time, while it was 0.8 for inter examiner reliability which indicated strong agreement between examiners [21].

## Methods

### Clinical procedures

**Fig. 2:**
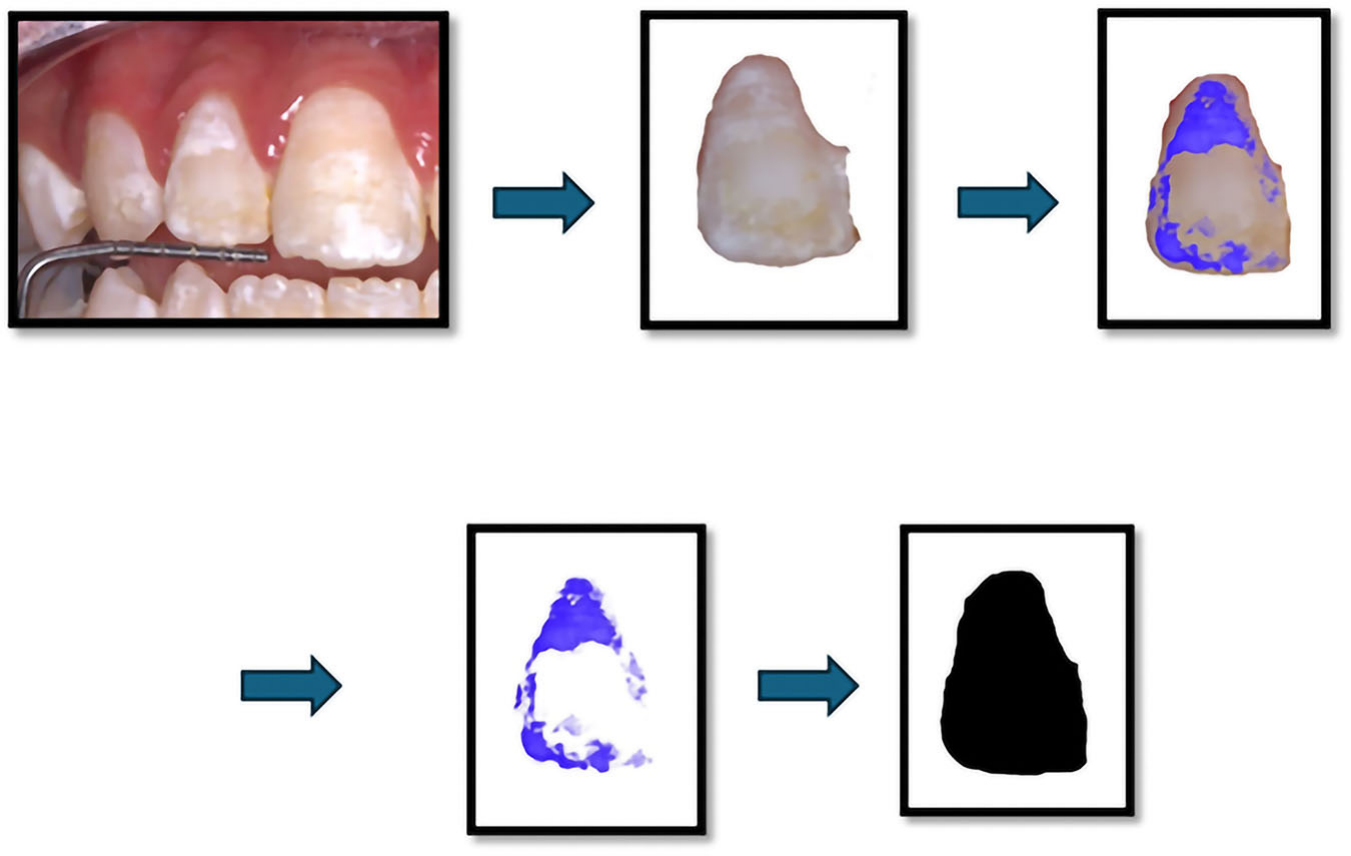
Steps of WSLs % calculation.

All intervention procedures were performed by the primary investigator as follows:

1. Dental prophylaxis was performed for all patients with a polishing brush and non-fluoridated prophylactic paste (PROPHY PASTE by PSP, universal grit, ENGLAND) during the first clinical examination and before any application to remove dental plaque [17].
2. For each tooth, baseline color change(ΔE) readings between normal tooth color and each WSL were performed by Vita Easyshade Spectrophotometer Compact (Vita Zahnfabrik, Bad Sa¨ckingen,Germany) [16].

- The device was calibrated prior to every visit with a white table provided by the manufacturer. Excess saliva was evacuated using an air syringe without causing undue desiccation. Each WSL was assessed by positioning the probe tip perpendicularly to the surface, and the measurements were documented. To reduce measurement mistakes, three consecutive measurements were obtained for each evaluated region, with the mean value calculated and used for subsequent analysis [25].
- The Model of the Commission International de l'Éclairage Lab (CIE L*a*b) color model was utilized to determine color variation. ΔE was computed using the subsequent equation: ΔE = √[(ΔL*)² + (Δa*)² + (Δb*)²], where: L* represents the lightness dimension (0 = black, 100 = white), a* indicates position on the red-green axis (positive = red, negative = green), b* denotes position on the yellow-blue axis (positive = yellow, negative = blue). Based on established perceptual thresholds, ΔE values ≤ 3.3 were considered clinically imperceptible color differences [26].

3) Photographic assessment was done at baseline, where all the photographs were taken with the digital camera, macro lens configuration and external flash source.

- The camera was configured in manual mode with an aperture setting of f/22 and a shutter speed of 1/125 s. The image quality was set to Fine, and the ISO sensitivity was fixed at 200. All photos were saved in JPEG (Joint Photographic Experts Group) format, ensuring compatibility with image analysis software for further processing.
- The clinical photographs were processed using photographic editing software (Adobe Photoshop 7.0, Adobe Systems Inc., San Jose, California, USA), then the WSL area was calculated as % of the total teeth area using Image J software (version 1.53a National Institutes of Health, USA). The image analysis steps, and measurement technique were performed as follows [27] (Fig. 2):

i. Step 1: The Photoshop software was used for the segmentation of each tooth by its outline, using the semiautomatic outline selection tool. In that way, each tooth was isolated from the rest of the image. After that, the areas with WSLs were automatically detected with the blue color range command and highlighted with blue and then separated from the rest of the image [28].
ii. Step 2: Using image j software, the entire visible tooth area was automatically measured in pixels. From the images of isolated WSL which separated in step 1 and applying a threshold, the WSL in each tooth was automatically measured in pixels, and thcalculated as % of the total teeth area using the following equation: [28]

$${{\rm{WSL}}} \% =\frac{{{\rm{Sum}}}.{{\rm{of}}}\; {{\rm{WSLstained}}}\; {{\rm{area}}}({{\rm{pixels}}})}{{{\rm{Total}}}\; {{\rm{tooth}}}\; {{\rm{area}}}({{\rm{pixels}}})}\times 100$$

4) Application of remineralizing agents

**Fig. 3:**
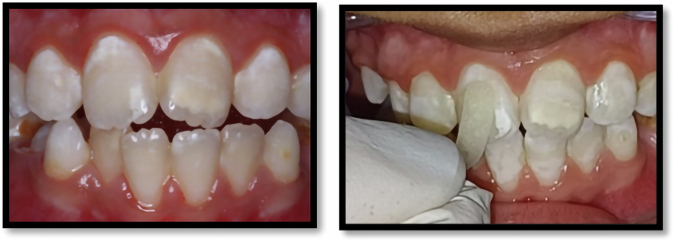
Application of (Self assembling peptide P11-4).

**Fig. 4:**
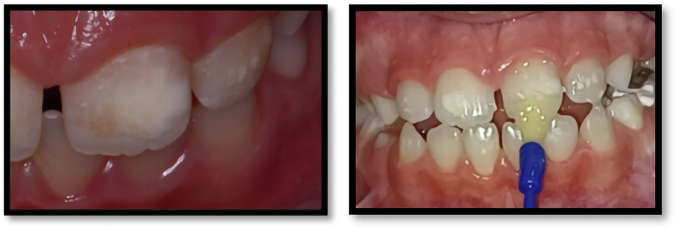
Application of Arginine Enriched Sodium fluoride group.

**Fig. 5:**
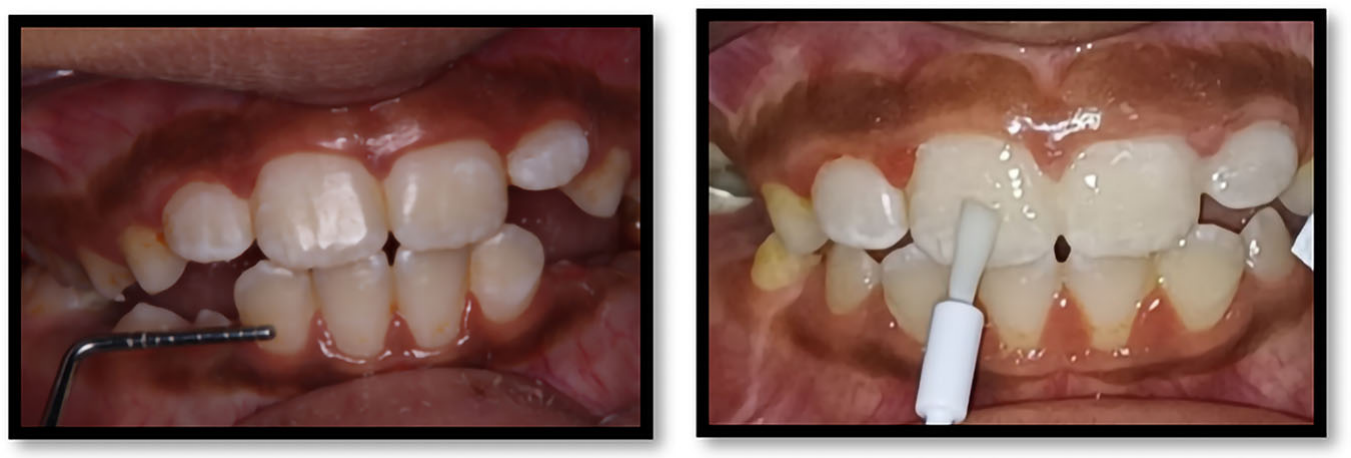
Application of Clinpro White Varnish.

A. **Group I (Self assembling peptide P11-4):** The lesions were treated with NaOCl to eliminate pellicle, etched for 20 s with 35% H_3_PO_4_ etching gel (BISCO SELECT, HV ETCH high viscosity) to partially demineralize tooth surface, and washed with water for 20 s. The lesions were dried using air tip syringe for 5 s and isolated using cotton rolls. The stick of self-assembling peptide (Curodont Repair TM, Credentis AG, Windisch, Switzerland) solution was inserted and subsequently withdrawn with its dampened sponge tip. Application of Curodont Repair ensued, allowing the solution to diffuse for five minutes until the tooth surface appeared dry (Fig. 3) [8].
B. **Group II (Arginine Enriched Sodium fluoride group):** The Arginine powder (L-arginine A5006, Sigma-Aldrich, St. Louis, MO, USA) was applied at 2% w/v with 5% sodium fluoride varnish (Citrine sodium fluoride varnish Dharma Research, Inc). This was achieved by mixing 200 mg of arginine with 10 ml of sodium fluoride varnish for 60 s using a sterile micro brush. WSLs were gently dried using air tip syringe. The prepared varnish was then applied evenly with a micro brush and allowed to remain undisturbed for five minutes (Fig. 4) [29, 30].
C. **Group III (Tri Calcium Phosphate fluoride):** TCP varnish (Clinpro white varnish, 3 M ESPE) was first mixed with the supplied brush to avoid the separation of the material during storage [31] A thin uniform layer of the varnish was applied on the lesion surface using the supplied micro brush (Fig. 5).

#### Post treatment instructions

- Patients were instructed to refrain from rinsing, consuming food, or drinking for the longest restriction period required among the applied materials (30 minutes for P11-4 [28], 2 h for arginine-enriched fluoride varnish [29], or immediate eating with food restrictions for TCP fluoride varnish [31]). All participants were advised to avoid hard, sticky, or hot foods for 4 h post-treatment. Cool water consumption was permitted immediately after application.
- All patients were advised to keep up proper dental hygiene by engaging in regular brushing.

#### Follw up

Patients were recalled for further applications after 3 and 6 months [8] and for WSLs assessment both photographic and colorimetric after 3, 6, 9 months followed CAMBRA guidelines for monitoring early caries intervention [8, 22].

### Statistical methods

Quantitative variables were reported as means ± standard deviations. Normality of distribution was verified using the Shapiro–Wilk test, while homogeneity of variance was assessed with Levene’s test. Parametric analysis of normally distributed color change data was achieved using a mixed-model analysis of variance (ANOVA). Post hoc pairwise comparisons were accomplished with Bonferroni-adjusted *p*-values to control for multiple comparisons. The alpha level for statistical significance was set a priori at *p* < 0.05. All analyses were done using R statistical software (version 4.4.0) [32, 33].

In photographic assessment, the percentage of change was calculated by the following equation:

$$\frac{{Baseline\; value}-{value\; after\; time}(t)}{{Baseline\; value}}X100$$

- *P*-value ≤ 0.05 was considered statistically significant (95% significance level), while *p*-value ≤ 0.001 was deemed highly statistically significant (99% significance level).

## Results

### Color change

#### Inter group comparison of color change

ΔE values for all test materials are summarized in (Table 1).

**Table 1:** Comparisons and summary statistics of color change (ΔE) in different groups.

Material	Color change (ΔE) (Mean ± SD)			*p*-value
Time	Group I	Group II	Group III	
Base line	16.92 ± 3.73^a^	18.49 ± 4.51^a^	17.48 ± 3.56^a^	0.115 ns
3 months	16.39 ± 3.04^b^	14.06 ± 4.46^a^	14.80 ± 3.11^a^	<0.001^*^
6 months	12.85 ± 3.23^b^	11.58 ± 2.77^a^	11.50 ± 3.28^a^	<0.001^*^
9 months	12.21 ± 3.03^b^	9.37 ± 3.79^a^	9.15 ± 2.74^a^	<0.001^*^

Values with distinct superscripts in the same horizontal row are significantly different.

^*^Indicates significance (*p* < 0.05), whereas ns denotes non-significance.

^a,b,c,d^Values with distinct superscript in the same horizontal row are significantly different.

Baseline ΔE measurements revealed no significant intergroup differences (*p* > 0.05). At 3-month evaluation, Group I demonstrated significantly higher ΔE values (indicating less color improvement) compared to Groups II and III (*p* < 0.05). Longitudinal analysis showed progressive color improvement in all groups at 6- and 9-month intervals, with Group I maintain significantly elevated ΔE values versus both groups (*p* < 0.001). No statistically significant differences were seen between Groups II and III at any timepoint (3/6/9 months).

#### Intragroup comparison of color change

Quantitative comparisons of color change (ΔE) at each timepoint are summarized in (Table 2).

**Table 2:** Comparisons and summary statistics of color change (ΔE) for different times.

Time	Color change (ΔE) (Mean ± SD)				*p*-value
Material	Base line	3 months	6 months	9 months	
Group I	16.92 ± 3.7^a^	16.39 ± 3.0^a^	12.85 ± 3.2^b^	12.21 ± 3^b^	<0.001^*^
Group II	18.49 ± 4.5^a^	14.06 ± 4.4^b^	11.58 ± 2.7^c^	9.37 ± 3.7^d^	<0.001^*^
Group III	17.48 ± 3.5^a^	14.80 ± 3.1^b^	11.50 ± 3.28^c^	9.15 ± 2.74^d^	<0.001^*^

Values with distinct superscripts in the same horizontal row are significantly different.

^*^Indicates significance (*p* < 0.05).

^a,b,c,d^Values with distinct superscript in the same horizontal row are significantly different.

In group I, there was a significant decrease in Mean ± SD of ΔE values at 6- and 9-months intervals compared to baseline and 3 months (*p* < 0.001). While in group II and group III a significant difference was found in ΔE (*p* < 0.001) among successive time intervals.

### Photographic assessment results

#### Assessment of the mean lesion percentage

##### Intra-group comparison of the percentage of white spot lesions:

Effect of the time interval on the percentage of white spot lesions under the same group type presented in Table 3.

**Table 3:** Mean ± SD, and intra-group comparison of Lesion percentage for the three studied groups at different time intervals.

	Group I	Group II	Group III
Baseline	47.43 ± 8.91^a^	34.19 ± 5.95^a^	41.04 ± 13.73^a^
After 3 months	41.08 ± 9.51^a,b^	25.43 ± 6.87^b^	33.49 ± 11.35^b^
After 6 months	34.67 ± 6.87^b,c^	19.36 ± 7.67^c^	27.38 ± 9.86^b,c^
After 9 months	27.93 ± 8.98^c^	14.98 ± 7.55^c^	22.32 ± 8.61^c^
*P*-value*	<0.001^HS^	<0.001^HS^	<0.001^HS^

Values with distinct superscripts in the same horizontal row are significantly different.

*Indicates significance.

^a,b,c,d^Values with distinct superscript in the same horizontal row are significantly different.

^hs^Highly significant (p < 0.001)

All three treatment groups demonstrated progressive and statistically significant reductions in white spot lesion (WSL) percentages over the 9-month study period (p < 0.001).

In Group I (P11-4), the mean WSL percentage decreased from 47.43 ± 8.91% at baseline to 41.08 ± 9.51% at 3 months, 34.67 ± 6.87% at 6 months, and 27.93 ± 8.98% at 9 months. Significant reductions were observed between baseline vs. 6 and 9 months (*p* < 0.05), while changes between 3 vs. 6 months and 6 vs. 9 months were not statistically significant.

Group II (Arginine-Fluoride) showed the most substantial improvement, declining from 34.19 ± 5.95% at baseline to 25.43 ± 6.87% at 3 months, 19.36 ± 7.67% at 6 months, and 14.98 ± 7.55% at 9 months. All time intervals demonstrated significant differences (*p* < 0.05) except between 6 and 9 months.

For Group III (TCP Fluoride), the mean WSL percentage reduced from 41.04 ± 13.73% at baseline to 33.49 ± 11.35% at 3 months, 27.38 ± 9.86% at 6 months, and 22.32 ± 8.61% at 9 months. Significant improvements were seen between baseline and all follow-up intervals (*p* < 0.05) and between 3 and 9 months (*p* < 0.05), while differences between 3 vs. 6 months and 6 vs. 9 months were not statistically significant.

#### Assessment of the change in lesion percentage after different follow-ups

##### Intra-group comparison of the change % of Lesion area:

Effect of the time interval on the change % of Lesion area under the same group presented in Table 4.

**Table 4:** Mean ± SD, and intra-group comparison of change in lesion percentage for the three groups studied under the different time intervals.

	Group I	Group II	Group III
Change % after 3 months	13.57 ± 10.19^c^	25.94 ± 12.85^c^	17.63 ± 10.69^c^
Change % after 6 months	26.63 ± 7.22^b^	43.28 ± 18.6^b^	32.88 ± 13.01^b^
Change % after 6 months	40.13 ± 16.76^a^	56.21 ± 19.12^a^	45 ± 12.27^a^
*P*-value**	<0.001^HS^	<0.001^HS^	<0.001^HS^

Values with distinct superscripts in the same horizontal row are significantly different.

*Indicates significance.

^a,b,c,d^Values with distinct superscript in the same horizontal row are significantly different.

^hs^Highly significant  (p<0.001)

All three treatment groups demonstrated progressive and statistically significant increases in lesion area reduction percentages over time (*p* < 0.001). Group I showed mean changes of 13.57 ± 10.19% at 3 months, 26.63 ± 7.22% at 6 months, and 40.13 ± 16.76% at 9 months. Group II exhibited the most substantial improvements with 25.94 ± 12.85%, 43.28 ± 18.6%, and 56.21 ± 19.12% reduction at respective intervals. Group III displayed intermediate results with 17.63 ± 10.69%, 32.88 ± 13.01%, and 45 ± 12.27% reduction.

#### Inter-group comparison of the change % of Lesion area

Effect of the group type on the change % of lesion area under the same time interval presented in Table 5.

**Table 5:** Mean ± SD, and inter-group comparison of the change in lesion percentage for the three groups studied at different follow ups.

	Group I	Group II	Group III	*P*-value*
After 3 months	13.57 ± 10.19^b^	25.94 ± 12.85^a^	17.63 ± 10.69^b^	<0.001^HS^
After 6 months	26.63 ± 7.22^b^	43.28 ± 18.6^a^	32.88 ± 13.01^b^	<0.001^HS^
After 9 months	40.13 ± 16.76^b^	56.21 ± 19.12^a^	45 ± 12.27^b^	0.004^S^

Values with distinct superscripts in the same horizontal row are significantly different.

*Indicates significance.

^a,b,c,d^Values with distinct superscript in the same horizontal row are significantly different.

^hs^Highly significant  (p<0.001)

^s^Significant

From Table 5, we can conclude the following:

- For all time intervals, Group II achieved the highest means of change% while Group I achieved the lowest mean.
- There was no significant difference between Group I and Group III means at all time intervals.
- Also, there was a significant difference between Group I and Group II as well as between Group II and Group III at all time intervals.

## Discussion

A white spot lesion is a visual phenomenon caused by the loss of subsurface tissue. The surface features of active WSLs display enlarged intercrystalline spaces and diminished interprismatic mineral content in the surface layer. The restorative treatment of first enamel lesions results in adverse effects on the dental structure. The objective of contemporary dentistry emphasizes noninvasive treatment of WSLs [23, 31].

Given the lack of reliable evidence supporting remineralizing or camouflaging strategies for treating white spot lesions, further well performed clinical studies are necessary to determine the most effective clinical methods [34].

This randomized controlled trial aimed to evaluate color improvement in white spot lesions (WSLs) following treatment with three remineralizing agents: a self-assembling peptide (P11-4), a 2% arginine-enriched sodium fluoride varnish, and a tri-calcium phosphate (TCP) fluoride varnish. Lesion shade was measured at baseline, 3, 6, and 9 months using a VITA Easyshade spectrophotometer. These materials have been shown to provide effective, minimally invasive treatment for early caries, offering superior results compared to traditional fluoride therapies [29, 35, 36].

The participants included were selected from an age range of 8–12 years as the prevalence of white spot lesions significantly escalates during the pre-adolescent stage. Moreover, younger patients exhibit greater susceptibility to remineralization compared to older individuals. No conclusive evidence exists regarding a sexual predisposition to WSLs, whereas the labial surfaces of maxillary anterior teeth accounted for 73% of WSLs. The participants were free from systemic illnesses or concurrent drugs that could reduce salivary flow rate [37]. Patients exhibiting developmental enamel defects such as hypomineralization disorders (e.g., fluorosis, hypoplasia) were not included in this research. These conditions involve genetic mutations that disrupt enamel matrix formation, making the affected tooth structure less responsive to remineralization therapies compared to typical caries lesions [24, 38].

White spot lesions were visually evaluated using the air dryness test, as it is considered the gold standard for assessing color changes and the depth of these lesions [24]. Prior to the assessment in the current study, dental prophylaxis was performed to prevent masking the tooth color and to ensure accurate readings [16].

Color evaluation was conducted utilizing a VITA Easyshade® guide spectrophotometer. Instrumental color analysis presents a distinct advantage over ocular color assessment due to its objective, quick, and quantifiable nature. VITA Easyshade® when compared to other spectrophotometers, is considered feasible, applicable and convenient with acceptable repeatability [39].

Dimensions of WSLs were assessed by digital photography as it was proven that standardized photographic technique is reliable, accurate, and reproducible compared to direct ICDAS II examination, confirming its effectiveness for assessing WSLs in digital images. The percentage area of WSL per total facial tooth surface was calculated to control magnification differences [19].

Digital cameras offer notable benefits, as highlighted by Benson et al. [27] by minimizing inconsistencies in image capture and improving efficiency. Quantitative assessment of enamel demineralization can be achieved by measuring either the dimensions of white spot lesions or the degree of mineral loss.

Digital camera canon D800 was used as it allows full flexibility regarding exposure controls and accessories, such as the use of a macro lens (sigma 108 mm) for close-up work and external flash ring units (Godox flash ring) to capture important differences in enamel color. This camera also produces high-resolution images [27].

Self-assembling peptide was implemented in accordance with the equipment manufacturer’s standardized operational guidelines. The rationale for treating enamel with 2% sodium hypochlorite is to remove surface organic pellicle, thus increasing the diffusion of Ca²⁺ and PO₄³⁻ ions into the enamel subsurface lesion. Teeth were conditioned with 35% Phosphoric acid for 20 s to remove the pseudo-calcified outermost 25 μm of the enamel surface which would increase surface porosity enhancing the remineralization process and allowing easier access for the minerals to the body of the lesion [40].

In present study, 2% by w/v arginine was used, because it was reported that combining 2% L-arginine into 5% NaF varnish enhances the physical characteristics of fluoride and creates a stable matrix that consistently releases greater levels of F/Arg compared to other concentrations [41].

Tri-calcium phosphate fluoride varnish used as positive control, was selected for its effectiveness as a fluoride-based remineralizing agent. The varnish facilitates remineralization by supplying calcium, phosphate, and fluoride ions, with a high fluoride concentration (22,600 ppm) allowing for a single application [31, 42, 43].

Follow up was performed at 3, 6, 9 months as the mineral deposition is expected to occur within 3–6 months [36]. Multiple applications were performed at different intervals 0, 3, 6 months as repeated applications performed ensures the consistent availability of ions to promote mineral growth. Over time, this leads to substantial repair and filling of the lesion. Another reason for multiple applications is that remineralization is not immediate and requires a consistent therapeutic effect over weeks or even months [36, 44, 45].

The findings of the current investigation indicated that all three evaluated materials demonstrated an enhancement in the hue of WSL. For color change (ΔE) measured at baseline, there was no statistically significant difference between different materials. At 3 months, the highest ΔE value was found (lowest color change) in group I (16.39 ± 3.04), which was significantly higher than group II (14.06 ± 4.46) and group III (14.80 ± 3.11). At 6 and 9 months, all the tested materials showed color improvement with the highest values in group I at 6 months (12.85 ± 3.23) and 9 months (12.21 ± 3.03) followed by group III at 6 months (11.50 ± 3.28) and 9 months (9.15 ± 2.74), while the lowest values were recorded in group II at 6 months (11.58 ± 2.77) and 9 months (9.37 ± 3.79).

In the clinical assessment of white spot lesions (WSLs), all tested materials reduced lesion percentage, but to varying degrees. Group II (2% arginine-enriched sodium fluoride varnish) showed the greatest reduction (14.98 ± 7.55), performing significantly better than the other groups. Group I (self-assembling peptide) had the highest lesion percentage (27.93 ± 8.98), followed by Group III (functionalized tricalcium phosphate fluoride varnish) (22.32 ± 8.61), with no significant difference between them. However, Group I differed significantly from Group II at all time points, highlighting the superior efficacy of the arginine-enriched fluoride varnish in reducing WSLs.

In group I, there was a significant decrease in Mean ± SD of ΔE values at 6- and 9-months intervals compared to baseline and 3 months (*p* < 0.001). While in group II and group III a significant difference was found in ΔE (*p* < 0.001) among successive time intervals.

The findings of this investigation suggested that group I (self-assembling peptide) improved the color of WSLs, but to a lesser extent than the other groups and this came in agreement with Wierichs et al. [46] who reported that P 11-4 could reduce lesion progression but did not significantly improve the visual appearance or mask the white spots as effectively as low-viscosity resin infiltrates.

These results also concurred with, Golland et al. [47] who showed that the application of P11-4 on demineralized bovine enamel did not result in increased fluorescence, as evaluated by quantitative laser fluorescence, suggesting either a lack of remineralization or the existence of irregular crystals. Additionally, Memarpour et al. [48] revealed that P11-4 peptide treatment resulted in primary teeth exhibiting the lowest percentage of surface enamel microhardness compared to CPP-ACP, fluoridated bioactive glass toothpaste, and fluoridated toothpaste. Recently, a study by Wahba et al. [49] reported the inability of P11-4 peptide to remineralize caries in deciduous teeth.

These results diverged with those of Alkilzy et al. [45] who reported superior clinical outcomes for P11-4 in managing early occlusal caries in pediatric patients. Their study demonstrated significantly reduced laser fluorescence measurements and improved visual assessment scores in the peptide-treated group versus fluoride controls. Furthermore, they observed caries regression (ICDAS index improvement) and lesion inactivation (Nyvad criteria), suggesting P11-4’s biomimetic properties combined with fluoride may enhance early caries reversal through noninvasive remineralization.

Jablonski-Momeni et al. [20] and Sedlakova Kondelova et al. [50] also reported the benefits of P11-4 in conjunction with fluoride compared to the application of fluoride varnish alone. Kamal et al. [40] also showed that the P11-4 peptide had enhanced efficacy when coupled with either fluoride or CCP-ACPF, compared to its solitary use.

The findings of this study indicated no significant improvement at 3-months- interval in group I. However, with multiple applications over a longer period (6 and 9 months), noticeable improvement was observed. This came compatible with Rathore et al. [44] and Vas et al. [51] who showed that significant mineral gains between 8 and 12 weeks after self-assembling peptide treatment occur, with increased effectiveness over longer intervals.

The results of group II (2% Arginine Enriched Sodium Fluoride varnish) showed higher improvement in color than other groups at all intervals. This could be attributed to its efficacy in halting caries progression, as arginine-fluoride complexes can be retained within enamel, serving as a reservoir that releases fluoride during acid exposure, thereby facilitating the remineralization process. Additionally, it elevates pH, establishing favorable conditions for remineralization and augmenting the synergistic effect of fluoride [41].

These results came in agreement with Bijle et al. [52] reported that 2% arginine-enhanced sodium fluoride toothpaste significantly improved enamel lesion remineralization rates. Similar outcomes were observed with Cheng et al. [10] and Oliveira et al. [53] whose studies on bovine enamel showed that the fluoride-arginine combination enhanced surface microhardness in both sound and demineralized specimens.

Furthermore, this came in line with Alblooshi et al. [30] who assessed the fluoride release potential of L-arginine incorporated into fluoride varnishes. Results showed that arginine significantly increased fluoride release compared to controls (Four readily available F varnishes), with the primary fluoride concentration being lower in the arginine groups, indicating a chemical interaction between fluoride and arginine. This suggests that adding arginine enhances the fluoride release potential, boosting the varnish’s effectiveness in remineralization and caries prevention.

Group III (Functionalized Tri Calcium Phosphate Fluoride varnish) showed improvement in the color of WSLs. This may result from fTCP’s capacity to function as a bioactive source of mineralizing constituents. TCP collaborates with fluoride to produce more robust, acid-resistant minerals compared to those formed by fluoride alone [14]. This result also came in agreement with Alamoudi et al. [14] reported significantly higher surface microhardness values for fluoride varnish containing functionalized tri-calcium phosphate (fTCP) compared to conventional fluoride formulations. Their study demonstrated mean Knoop hardness numbers (KHN) of 56.2 for 5% NaF+fTCP versus 45.7 for NaF alone, with untreated controls showing the lowest hardness (35.1 KHN).

Likewise, Rirattanapong et al. [54] also showed that fluoride varnish containing tricalcium phosphate had a high remineralization effect and can inhibit progression of WSLs. Elkassas and Arafa [55] found that remineralizing agents containing different calcium-phosphate formulas and fluoride have increased remineralization potential compared to artificial saliva. TCP varnish presented the highest remineralization tendency with the greatest resistance for acid challenge when compared to Tooth Mousse Plus and Vanish™XT.

None of the evaluated materials produced a color alteration in the white spot lesions (WSLs) that matched that of normal teeth as clinically tolerable color changes, deemed not visually noticeable, arise when ΔE* ≤3.3 which was not evident in any of the three groups [26].

The result of this study rejected the null hypothesis, demonstrating a substantial difference in color change (ΔE) across the investigated materials. The study designed as a randomized controlled double-blinded approach, reduces bias and improves the dependability of the findings. Moreover, the use of three distinct remineralizing agents facilitates a thorough comparison of efficacy in treating white spot lesions (WSLs). The evaluations at 3, 6, and 9 months yield significant insights into the long-term effect of the treatments.

On the other hand, the Vita Easy Shade Spectrophotometer primarily measures surface color and does not provide information on the depth of theWSL, which could be an important factor for treatment evaluation. The study focuses on color change as the primary outcome, potentially overlooking other important aspects of WSL improvement, such as surface integrity or depth of remineralization.

## Conclusions

All three examined materials demonstrated an enhancement in the coloration of WSLs. However, 2% Arginine Enriched Sodium Fluoride exhibited the most favorable outcomes, succeeded by Functionalized Tri Calcium Phosphate Fluoride varnish, whilst self-assembling peptide P11-4 demonstrated the least enhancement in color.

## Supplementary information

SI Table 1

## Data Availability

The datasets generated and/or analyzed during the current study are available from the first author “Bardis Salah” upon request.
