# Supplementary material for: The remineralizing ability of self-assembling peptide P11-4, 2% arginine enriched sodium fluoride and functionalized tri calcium phosphate fluoride varnishes in treatment of white spot lesions – a randomized controlled trial
Source: BDJ Open. 2025 Jul 27;11:70. doi: 10.1038/s41405-025-00353-5 (PMC12297324; doi:10.1038/s41405-025-00353-5)
Supplement: Supplementary file 1 — SI Table 1 [file 41405_2025_353_MOESM1_ESM.docx]

**SI Table 1: CAMBRA-Based Eligibility Criteria.**

| **Parameter** | **Inclusion Criteria** | **Exclusion Criteria** | **CAMBRA Rationale** |
| --- | --- | --- | --- |
| **Disease Indicators** | • ICDAS 1–2 WSLs on maxillary anteriors | • Cavitated lesions (ICDAS ≥3) | Active non-cavitated lesions = moderate risk |
| **Biological Risk Factors** |  |  |  |
| - Microbial Load: S. mutans\ Lactobacilli by CRT® Bacteria chair side Test | (<10⁵ CFU/mL S. mutans). | >10⁶ CFU/mL S. mutans + high Lactobacilli | High S. mutans = high risk |
| - Salivary Function: Stimulated salivary flow was measured by having participants chew paraffin wax for 5 minutes. Saliva was collected in pre-weighed tubes every 30 seconds. | • Normal stimulated flow (>0.7 mL/min) | • Xerostomia or hyposalivation | Low saliva = high risk |
| - Dietary Habits | • No frequent sugar intake (implied by exclusion) | • Sugar exposure >3x/day | Frequent sugar = high risk |
| - Plaque Control | • Prophylaxis before treatment | • Visible plaque accumulation (if severe) | Poor hygiene = high risk |
| **Protective Factors** |  |  |  |
| - Fluoride Use | • all patients drinking tape water that contain flouride | • Regular use of high-fluoride products | Home fluoride = protective |
| **General Factors** | • Age 8–12 years | • Systemic diseases/medications affecting caries risk | Controls for confounders |
|  | •Cooperative behavior | •Malocclusion/mouth breathing | Ensures standardized application |
